# Structure of recombinant *Thermoascus aurantiacus* xylanase TaXyn10 with an authentic N-terminal pyroglutamate

**DOI:** 10.1107/S2053230X26009155

**Published:** 2026-09-22

**Authors:** Jelena Mijatovic, Uwe Mueller, Manfred S. Weiss, Frank Lennartz

**Affiliations:** a Macromolecular Crystallography Helmholtz-Zentrum Berlin für Materialien und Energie Albert-Einstein-Strasse 15 12489Berlin Germany; University of Oxford, United Kingdom

**Keywords:** X-ray crystallography, xylanases, pyroglutamate, split-intein purification system

## Abstract

The thermostable GH10 xylanase from *Thermoascus aurantiacus* (TaXyn10) is an industrially relevant enzyme whose native structure contains an N-terminal pyroglutamate (PCA) modification. Recombinant production of proteins containing an N-terminal PCA is often hindered by purification strategies that disrupt the authentic N-terminus. Here, we report the 1.1 Å resolution crystal structure of TaXyn10 recombinantly produced in *Escherichia coli* and purified using a split-intein-based system. The structure closely resembles that of the native protein. Electron-density maps support the presence of an intact N-terminal PCA, with partial occupancy consistent with spontaneous cyclization of the exposed N-terminal glutamine after intein cleavage. These results demonstrate that split-intein-based purification enables recovery of the native mature state of TaXyn10 and can preserve physiologically relevant N-terminal PCA formation in recombinant protein produced in *E. coli*.

## Abstract 2

The high-resolution structure of the *T. aurantiacus* xylanase TaXyn10 expressed in *Escherichia coli* shows that recombinant production combined with split-intein-based purification recovers the native fold of TaXyn10 and permits the formation of an N-terminal pyroglutamate. These results demonstrate that this approach can enable recombinant proteins to adopt their native mature state in *E. coli*, including physiologically relevant post-translational modifications such as N-terminal pyroglutamate formation.

## 1. Introduction

Xylanases are a class of enzymes found in a broad range of microorganisms, including bacteria and fungi. They hydrolyze xylan, a hemicellulose polymer found in the cell walls of plants that is one of the most abundant biopolymers on Earth. The backbone of xylan consists of β-1,4-linked xylose residues that can be further decorated with substituents such as arabinofuranose. Their ability to cleave this glycosidic bond makes xylanases important in industrial applications ranging from bio-bleaching in paper manufacturing to biofuel production (Pavlova *et al.*, 2026 ▸). They can be divided into exoxylanases, which cleave from the end of xylan chains, and endoxylanases, which cleave internal bonds within the chain. Among the latter, GH10 and GH11 xylanases are widely used in industry. Members of the GH10 family are characterized by a broader substrate specificity, allowing them to degrade substituted xylans commonly found in plants and making them highly useful for the breakdown of plant biomass (Pavlova *et al.*, 2026 ▸).

In addition to their functional importance, GH10 xylanases have been extensively characterized structurally, and crystal structures of bacterial and fungal xylanases have provided insight into substrate binding and catalysis. One example of this is the set of structures of the GH10 family endo-1,4-β-xylanase from the thermophilic ascomycete *Thermoascus aurantiacus* (TaXyn10). TaXyn10 is a thermostable enzyme that retains activity at temperatures as high as 80°C (Tan *et al.*, 1987 ▸). Following secretion signal peptide cleavage, the mature ectodomain is a 302-amino-acid xylanase that adopts a typical (β/α)_8_-triosephosphate isomerase (TIM) barrel fold, consisting of eight α-helices and eight β-strands that form a barrel-like structure (Natesh *et al.*, 1999 ▸; Lo Leggio *et al.*, 1999 ▸). Early structural studies have shown that the active site consists of two glutamate residues that are located at the C-terminal end of the TIM barrel, and have proposed structural elements such as tight hydrophobic packing, salt bridges and a disulfide bond as important factors for the thermostability of TaXyn10 (Natesh *et al.*, 1999 ▸). Later studies revealed that substrate binding by TaXyn10 involves the stabilization of flexible residues that surround the active site and that sugar decorations, such as arabinofuranose, attached to the xylan backbone can increase the affinity of TaXyn10 towards such decorated substrates (Lo Leggio *et al.*, 2001 ▸; Vardakou *et al.*, 2005 ▸).

Structural studies have also shown that TaXyn10 has a pyroglutamate (PCA) at its N-terminus (Natesh *et al.*, 2003 ▸, 1999 ▸; Lo Leggio *et al.*, 1999 ▸, 2001 ▸), a post-translational modification that occurs in animals, plants and fungi but is also found in proteins from prokaryotes (Bochtler *et al.*, 2018 ▸). PCA forms through the intramolecular cyclization of an N-terminal glutamine or, in rarer cases, glutamic acid, in which the α-amino group attacks the side-chain amide carbonyl, leading to the formation of a five-membered lactam ring. This reaction can occur spontaneously under physiological conditions and is significantly faster for glutamine than for glutamic acid (Schilling *et al.*, 2008 ▸). In addition, in both eukaryotes and prokaryotes, this cyclization is often mediated by glutaminyl cyclases, enzymes predominantly located in the secretory pathway that drastically increase the rate of PCA cyclization compared with the spontaneous reaction (Seifert *et al.*, 2009 ▸; Xu *et al.*, 2021 ▸). N-terminal PCA has numerous functions, including protection from proteolytic degradation by aminopeptidases, stabilization of secondary structures or contributing to the active conformation of proteins (Hinke *et al.*, 2000 ▸; Van Coillie *et al.*, 1998 ▸; Lou *et al.*, 2006 ▸). In addition, PCA can also stabilize a protein and enhance its thermostability, examples of which include lignocellulolytic enzymes isolated from fungi (Wu *et al.*, 2017 ▸), although for TaXyn10 such a stabilizing effect of PCA has not been shown to date.

This variety of functions highlights the importance of PCA, and recombinant production strategies for proteins with naturally occurring N-terminal PCA, such as TaXyn10, need to be designed to allow its formation. However, N-terminal sequences such as an uncleaved starter methionine or residues left from the cleavage of purification tags with commonly used enzymes such as Tobacco etch virus or PreScission protease block a potential N-terminal glutamine or glutamic acid, thereby preventing PCA cyclization. One approach to circumvent this problem is the use of self-cleaving tags which generate an intact native N-terminus after cleavage, thereby permitting PCA formation.

Here, we report the 1.1 Å resolution crystal structure of TaXyn10, recombinantly produced in *Escherichia coli* and purified using a self-cleaving, split-intein-based system. We show that TaXyn10 produced using this system preserves the native mature structural state and contains an N-terminal PCA.

## 2. Materials and methods

### 2.1. Macromolecule production

The gene encoding TaXyn10 (residues 27–328, GenBank Accession No. AAF24127.1) with an N-terminal Protein Select split-intein affinity tag was synthesized and codon-optimized for expression in *E. coli* by GeneArt (Life Technologies; see Table 1 ▸). The gene was cloned into the pET-15b expression vector between the NcoI and XhoI restriction sites. TaXyn10 was recombinantly produced in SHuffle T7 *E. coli* cells (New England Biolabs) grown in Terrific Broth medium at 30°C. Protein expression was induced when the cells reached an optical density (OD_600_) of 0.6 by the addition of isopropyl β-d-1-thiogalactopyranoside (IPTG) to a final concentration of 0.5 m*M*. After induction, the temperature was lowered to 18°C and the cells were further incubated for 16 h. The cells were then harvested by centrifugation, resuspended in buffer consisting of 300 m*M* NaCl, 20 m*M* Tris–HCl pH 8.0 and lysed by sonication. After an additional centrifugation step, the supernatant was loaded onto a Protein Select column (Cytiva), incubated for 24 h at 20°C for tag cleavage and eluted with elution buffer consisting of 200 m*M* NaCl, 20 m*M* Tris–HCl pH 8.0. Since only a small fraction of the protein was cleaved after this first incubation, as determined by molecular-weight shift in SDS–PAGE, TaXyn10 was further purified by size-exclusion chromatography (SEC) using a Superdex S75 16/60 column (Cytiva) equilibrated with SEC buffer consisting of 150 m*M* NaCl, 10 m*M* Tris–HCl pH 8.0. Fractions containing pure TaXyn10 were then pooled, loaded onto a Protein Select column equilibrated with SEC buffer and incubated for 48 h at 20°C for tag cleavage. The protein was eluted with SEC buffer and tag cleavage was verified by SDS–PAGE. Fractions containing pure TaXyn10 were pooled, concentrated to 22.19 mg ml^−1^, flash-frozen in liquid nitrogen and stored at −80°C.

### 2.2. Crystallization

Purified TaXyn10 was screened for crystallization at 293 K using a grid screen based on earlier experiments with a differently tagged TaXyn10 construct recombinantly produced in *E. coli*. By screening the JCSG Core Suite, these experiments identified MES as a suitable buffer and ammonium sulfate as a suitable precipitant (Schnapka, 2019 ▸). To establish optimized crystallization conditions for TaXyn10 purified with the split-intein-based system used here, the pH of MES was varied in steps of 0.5 between 5.7 and 6.7 and the ammonium sulfate concentration was varied in 0.2 *M* steps from 1.0 to 2.4 *M*. Plate-like crystals grew in 100 m*M* MES pH 6.7, 1.2 *M* ammonium sulfate after one week. These crystals were harvested, transferred into reservoir solution containing 20%(*v*/*v*) glycerol as a cryoprotectant and flash-cooled in liquid nitrogen for storage. Crystallization conditions are summarized in Table 2 ▸.

### 2.3. Data collection and processing

Data were collected on the BESSY II macromolecular crystallography beamline BL14.1 (Mueller *et al.*, 2012 ▸, 2015 ▸, 2025 ▸) at a wavelength of 0.918 Å using an MD2 microdiffractometer and a PILATUS3 X 6M detector. Samples were cooled to 100 K in a nitrogen gas stream. The data were processed with *XDSAPP* (Sparta *et al.*, 2016 ▸) and *AIMLESS* (Agirre *et al.*, 2023 ▸). The data-collection and processing statistics are summarized in Table 3 ▸. The crystal displayed a relatively low solvent content of 35.1% (Matthews coefficient *V*_M_ = 1.92 Å^3^ Da^−1^), which is consistent with the low overall Wilson *B* factor of 4.3 Å^2^ obtained during data processing with *AIMLESS*.

### 2.4. Structure solution and refinement

The crystal structure of TaXyn10 recombinantly produced in *E. coli* was solved by molecular replacement with *Phaser-MR* (McCoy *et al.*, 2007 ▸), using the crystal structure of TaXyn10 isolated from *T. aurantiacus* (PDB entry 1gok; Lo Leggio *et al.*, 2001 ▸) with its N-terminal PCA residue removed as an input model. The model was rebuilt by iterative cycles of model building in *Coot* (Emsley *et al.*, 2010 ▸) and refinement with *REFMAC*5 (Murshudov *et al.*, 2011 ▸), using individual anisotropic *B*-factor refinement in combination with automatically generated translation/libration/screw restraints and H atoms added at riding positions during refinement. The occupancy of PCA2 or Gln2 was refined independently using *REFMAC*5, without applying occupancy restraints or constraints. The refinement statistics are shown in Table 4 ▸. A composite omit electron-density map was generated with *phenix.refine* (Liebschner *et al.*, 2019 ▸) using a model in which the N-terminal PCA2 had been removed and which was subsequently refined with *REFMAC*5 as described above. Real-space *R* values and real-space correlation coefficients were calculated using *EDSTAT* (Tickle, 2012 ▸). Residual peak heights were determined with *Coot*. All structure figures were generated with *PyMOL* (Schrödinger).

## 3. Results

### 3.1. Structure of TaXyn10 produced in *E. coli*

To facilitate the formation of the single disulfide bond in TaXyn10, the protein was recombinantly produced in T7 SHuffle *E. coli* cells. After purification via split-intein-based affinity chromatography, the protein was further purified by size-exclusion chromatography, followed by tag cleavage. Complete tag cleavage was confirmed by size shift on SDS–PAGE. Recombinant TaXyn10 crystallized in space group *P*2_1_, and crystals diffracted to 1.11 Å resolution with one copy of TaXyn10 in the asymmetric unit, yielding a Matthews coefficient of 1.92 Å^3^ Da^−1^, corresponding to a solvent content of 35.1%.

**Figure 1:**
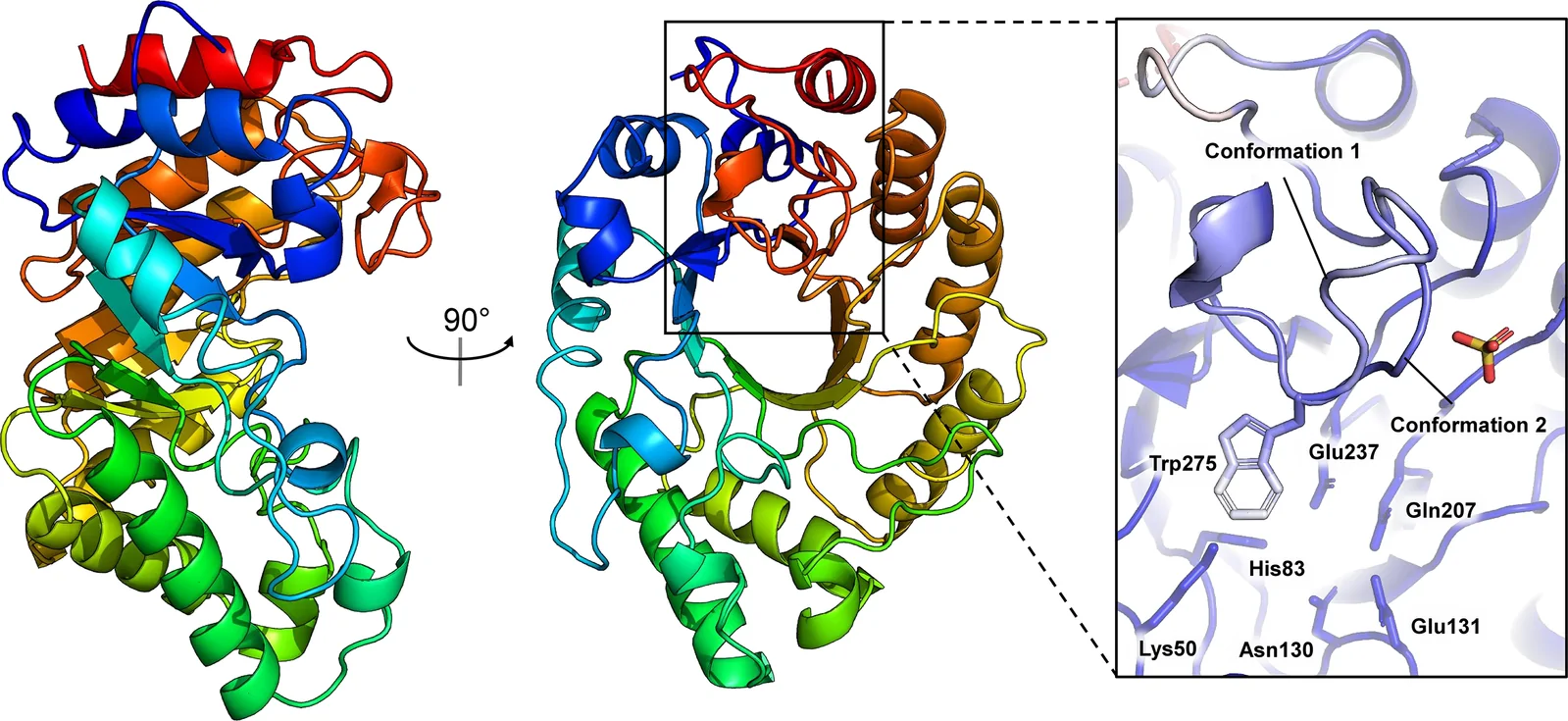
Structure of recombinantly produced TaXyn10. TaXyn10 is shown as a cartoon in rainbow representation, with the N-terminus coloured blue and the C-terminus coloured red. The inset shows the two modelled conformations for residues 276–280, coloured by *B* factors, with blue representing the minimum and red representing the maximum *B* factor for the protein chain. Trp275 and other residues important for substrate binding are shown in stick representation and a sulfate ion next to the flexible loop is shown in stick representation and coloured by element.

The overall structure of recombinant TaXyn10 is very similar to that of the protein isolated from *T. aurantiacus*, adopting the typical TIM-barrel fold (Fig. 1 ▸), with an r.m.s.d. of 0.26 Å over 297 C^α^ atoms and an r.m.s.d. of 0.65 Å over 2330 protein atoms compared with the high-resolution structure of TaXyn10 (PDB entry 1i1w; Natesh *et al.*, 2003 ▸), excluding a flexible loop comprising residues 276–280 in recombinant TaXyn10. Features that are thought to contribute to the thermostability of TaXyn10, such as the disulfide bond and an extensive salt-bridge network that includes a buried salt bridge between Arg150 and Glu258 (Natesh *et al.*, 2003 ▸, 1999 ▸; Lo Leggio *et al.*, 2001 ▸), were also observed. In contrast to earlier structures, residues 302–305 that form part of the eight βα-loop can be modelled in two alternate conformations, with elevated *B* factors relative to the rest of the structure, indicating flexibility (Fig. 1 ▸). Of note, a sulfate ion from the crystallization solution interacts with this loop in both conformations, which could have a stabilizing effect that captures these distinct conformations. Interestingly, this part of the loop is directly adjacent to Trp301, which in unliganded structures has been proposed to be more flexible and becomes ordered upon substrate binding (Lo Leggio *et al.*, 2001 ▸). The conformational heterogeneity observed here is therefore consistent with a model of a flexible region surrounding Trp301 that becomes stabilized upon ligand binding.

**Figure 2:**
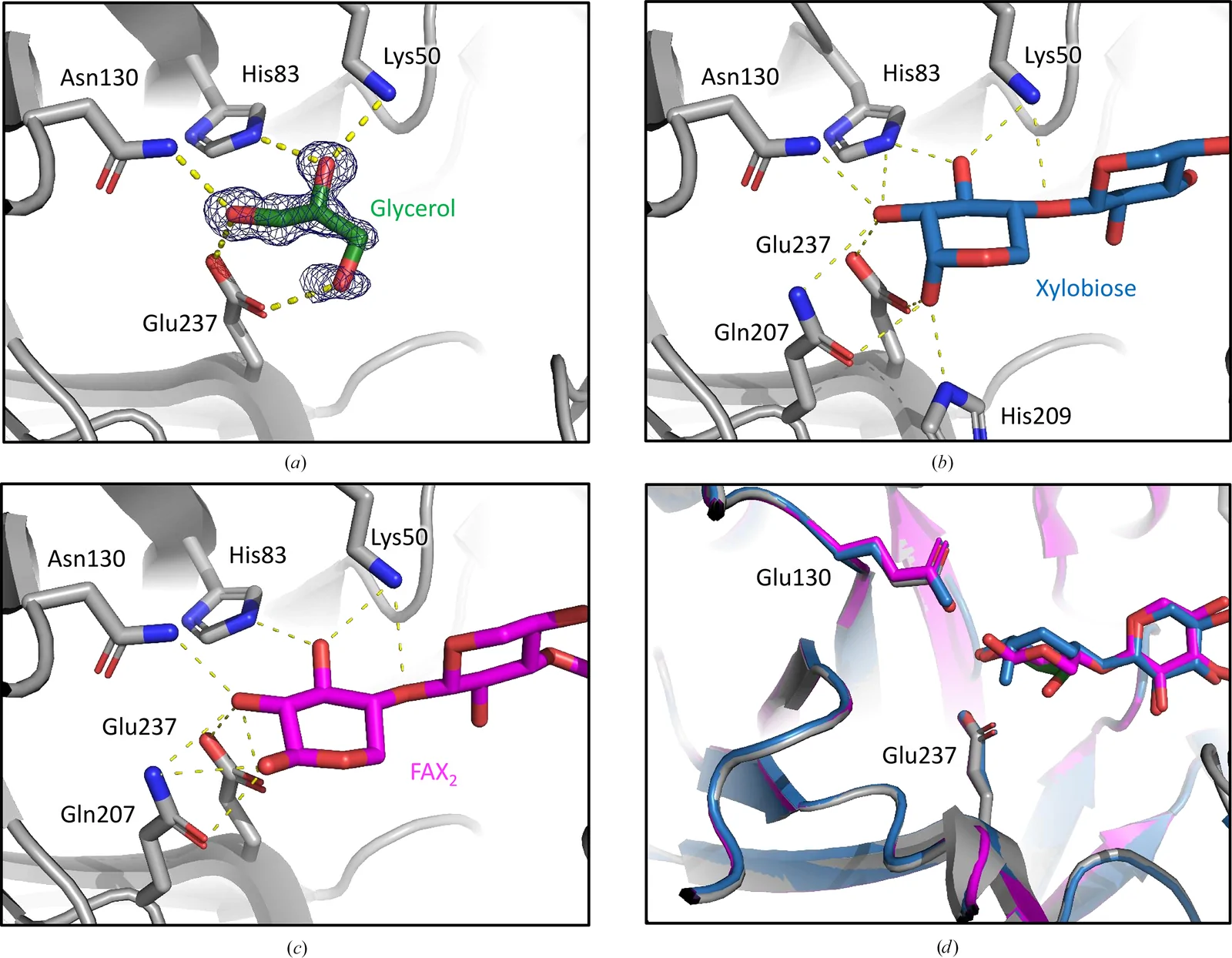
Structure of TaXyn10 subsite −1 bound to (*a*) glycerol, (*b*) xylobiose (PDB entry 1gor) or (*c*) xylobiose linked to feruloyl-arabinose (FAX_2_; PDB entry 2bnj). TaXyn10 is shown as a cartoon and coloured grey, interacting residues and the bound molecules are shown as sticks, and hydrogen bonds are indicated as yellow dotted lines. Glycerol is coloured green, xylobiose blue and FAX_2_ magenta. For the glycerol molecule, the 2*mF*_o_ − *DF*_c_ density contoured at 1.0s is shown as blue mesh. (*d*) An overlay of subsite −1 for TaXyn10 bound to glycerol, xylobiose or FAX_2_. Glycerol-, xylobiose- and FAX_2_-bound TaXyn10 are shown in grey, blue and magenta, respectively. The catalytic residues Glu130 and Glu237 are shown as sticks.

Furthermore, a glycerol molecule from the cryoprotectant solution was bound in subsite −1 of the active site, forming hydrogen bonds to residues Lys50, His83, Asn130 and Glu237 (Fig. 2 ▸*a*). This glycerol molecule partly mimics the interactions observed between TaXyn10 and the substrate xylobiose (Lo Leggio *et al.*, 2001 ▸; Fig. 2 ▸*b*) or xylobiose linked to feruloyl-arabinose (Vardakou *et al.*, 2005 ▸) in this subsite. However, xylobiose also forms hydrogen bonds to Gln207 and His209 (Fig. 2 ▸*b*), whereas the feruloyl-arabinose-linked xylobiose also contacts Gln207 (Fig. 2 ▸*b*). None of these interactions are observed in the glycerol-bound structure of TaXyn10. In addition, glycerol binding in subsite −1 does not lead to any conformational changes for the catalytic glutamates Glu130 and Glu237 (Fig. 2 ▸*d*).

### 3.2. Spontaneous N-terminal PCA formation in recombinantly produced TaXyn10

**Figure 3:**
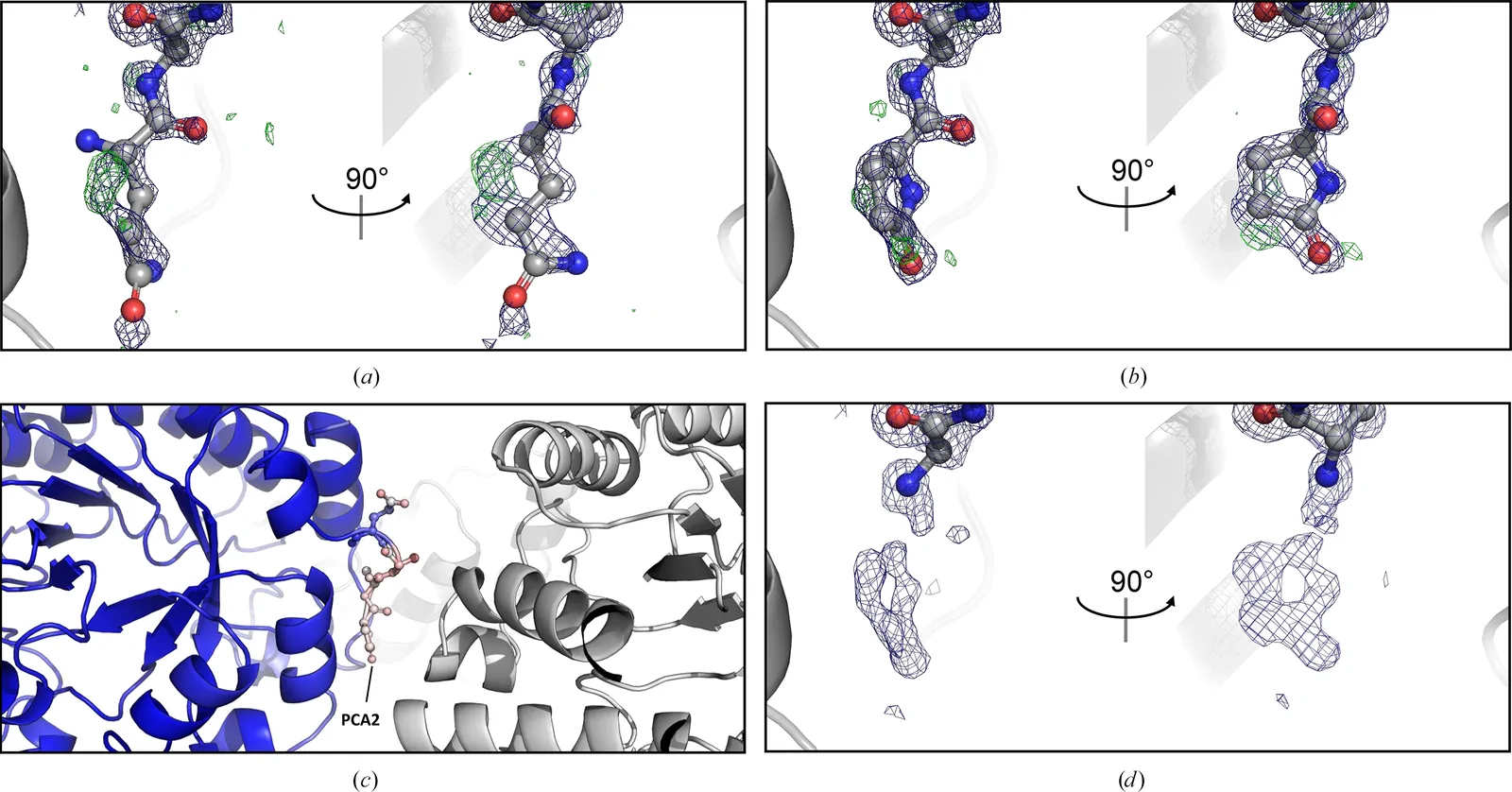
Electron density around the N-terminal PCA in recombinant TaXyn10, calculated after final refinement of the model. (*a)* Shown are the 2*mF*_o_ − *DF*_c_ electron-density maps (blue mesh) contoured at 1.0σ and the *mF*_o_ − *DF*_c_ difference electron-density maps (green and red mesh) contoured at 3.0σ around the N-terminal residue modelled as Gln. (*b*) 2*mF*_o_ − *DF*_c_ electron-density maps (blue mesh) contoured at 1.0σ and *mF*_o_ − *DF*_c_ difference electron-density maps (green and red mesh) contoured at 3.0σ around the N-terminal residue modelled as PCA. (*c*) TaXyn10 shown as a cartoon, coloured by *B* factor with blue representing the minimum and red representing the maximum *B* factor for the protein chain. The four N-terminal residues, including PCA2, are shown in ball-and-stick representation. Symmetry mates are shown as grey cartoons. (*d*) 2*mF*_o_ − *DF*_c_ composite omit electron-density map (blue mesh) contoured at 0.7σ around the N-terminal PCA.

Refinement of the TaXyn10 model with an N-terminal glutamine resulted in positive difference electron density, indicating that the N-terminal density cannot be explained by glutamine alone (Fig. 3 ▸*a*). Inspection of the 2*mF*_o_ − *DF*_c_ electron density around this N-terminal glutamine indicated a ring-like structure into which PCA could be modelled (Fig. 3 ▸*b*). Of note, the occupancy of PCA refined to 0.64, with a *B* factor of 12.85, which is similar to the subsequent alanine residues Ala3 and Ala4, which have *B* factors of 13.07 and 13.68 Å^2^, respectively. This partial occupancy is consistent with incomplete conversion of the N-terminal glutamine to PCA after cleavage of the tag. Indeed, independent refinement of a model with the N-terminal glutamine at full occupancy and a model with PCA with an occupancy of 0.64 showed that a glutamine at full occupancy leads to a higher real-space *R* value (0.218 versus 0.112) and a lower real-space correlation coefficient (0.70 versus 0.85) as well as stronger positive and negative *mF*_o_ − *DF*_c_ difference-density peaks around the residue. The N-terminal 2*mF*_o_ − *DF*_c_ density is only clearly visible when contoured at 1σ, which can partly be explained by the fact that the N-terminus points towards a solvent channel, resulting in higher flexibility as indicated by elevated *B* factors of the N-terminal residues compared with the rest of the model (Fig. 3 ▸*c*). Furthermore, a composite omit electron-density map calculated for a model which was refined without the N-terminal PCA showed weak ring-like electron density at the N-terminus (Fig. 3 ▸*d*) that is consistent with a PCA moiety, providing additional support for the interpretation of the N-terminal residue as PCA.

## 4. Discussion

Here, we report the X-ray crystallographic structure of TaXyn10 recombinantly produced in *E. coli* and identify the presence of an N-terminal PCA. This is the first structural study on recombinantly produced TaXyn10, and the low r.m.s.d. to the structure of protein isolated from native sources (Natesh *et al.*, 2003 ▸) shows that expression of TaXyn10 in the oxidizing cytoplasm of SHuffle T7 *E. coli*, which promotes disulfide-bond formation, preserves the overall native fold. While the oxidizing environment could influence protein folding, the close structural agreement indicates that it does not substantially alter the overall structure of TaXyn10. Importantly, the structure obtained here also contains the N-terminal PCA, providing structural evidence for PCA formation in recombinantly produced TaXyn10. N-terminal PCA formation is associated with enhanced thermal and proteolytic stability in industrially relevant lignocellulolytic enzymes (Wu *et al.*, 2017 ▸), such as the xylanase studied here. For cyclization to PCA, the N-terminal glutamine or glutamic acid needs to be freely available, which often is not the case for proteins produced in *E. coli* due to N-terminal residues left over from purification-tag cleavage or incomplete removal of the N-terminal methionine. Our results indicate that the strategy to use a self-cleaving split-intein-based system for purification facilitates PCA formation, which can be relevant to enhance the stability of recombinantly produced proteins such as TaXyn10. Since the N-terminal glutamine is blocked by the split-intein tag before cleavage, the PCA observed in the structure presented here has likely formed spontaneously. This is further supported by the partial occupancy of PCA, which could indicate that a fraction of the molecules in the crystal retain the N-terminal glutamine. Further biochemical experiments could establish the time-course of this N-terminal PCA formation and determine whether a longer incubation after tag cleavage could result in a higher proportion of N-terminal PCA. Alternatively, expression or purification strategies that use glutaminyl cyclases could result in higher cyclization rates.

In addition, TaXyn10 produced in *E. coli* using this split-intein-based system crystallized within a week and forms relatively robust plate-like crystals. These properties, together with the spontaneous formation of an N-terminal PCA modification that is readily observable in the electron density, make TaXyn10 an attractive model system for practical courses in X-ray crystallography, as it combines straight­forward crystallization behaviour with an instructive example for analysing a post-translational modification and the concept of partial occupancy.

## Supplementary Material

PDB reference: *Thermoascus aurantiacus*xylanase TaXyn10, 31ct

## Figures and Tables

**Table 1:** Macromolecule-production information

Source organism	*Thermoascus aurantiacus*
DNA source	Synthetic
Expression vector	pET-15b
Expression host	*Escherichia coli*
Complete amino-acid sequence of the construct produced	VKIVSRKSLGVQNVYDIGVEKDHNFLLANGLIASN**Q**†AAQSVDQLIKARGKVYFGVATDQNRLTTGKNAAIIQADFGQVTPENSMKWDATEPSQGNFNFAGADYLVNWAQQNGKLIRGHTLVWHSQLPSWVSSITDKNTLTNVMKNHITTLMTRYKGKIRAWDVVNEAFNEDGSLRQTVFLNVIGEDYIPIAFQTARAADPNAKLYINDYNLDSASYPKTQAIVNRVKQWRAAGVPIDGIGSQTHLSAGQGAGVLQALPLLASAGTPEVAITELDVAGASPTDYVNVVNACLNVQSCVGITVWGVADPDSWRASTTPLLFDGNFNPKPAYNAIVQDLQ

† N-terminal glutamine after split-intein tag cleavage.

**Table 2:** Crystallization

Method	Vapour diffusion, sitting drop
Plate type	48-well plate
Temperature (K)	293
Protein concentration (mg ml^−1^)	22.19
Buffer composition of protein solution	150 m*M* NaCl, 10 m*M* Tris–HCl pH 8.0
Composition of reservoir solution	100 m*M* MES pH 6.7, 1.2 *M* ammonium sulfate
Volume and ratio of drop	0.5 µl protein solution + 0.5 µl reservoir solution
Volume of reservoir (µl)	200

**Table 3:** Data collection and processing Values in parentheses are for the outer shell.

Diffraction source	BL14.1, BESSY, Berlin
Wavelength (Å)	0.918
Temperature (K)	100
Detector	PILATUS3 X 6M
Crystal-to-detector distance (mm)	150
Rotation range per image (°)	0.1
Total rotation range (°)	360
Exposure time per image (s)	0.1
Space group	*P*2_1_
*a*, *b*, *c* (Å)	45.53, 58.35, 50.71
α, β, γ (°)	90.00, 110.79, 90.00
Mosaicity (°)	0.25
Resolution range (Å)	42.57–1.11 (1.13–1.11)
Total No. of reflections	641004
No. of unique reflections	97755
Completeness (%)	99.9 (99.7)
Multiplicity	6.6 (6.5)
〈*I*/σ(*I*)〉	9.0 (1.9†)
CC_1/2_	99.7 (47.7)
*R*_r.i.m._ (%)	0.083 (0.416)
Overall *B* factor from Wilson plot (Å^2^)	4.3

† Meaningful signal, as judged by CC_1/2_, was observable below a mean *I*/σ(*I*) of 2.0 in the outer shell. The resolution at which mean *I*/σ(*I*) falls below 2.0 is 1.14 Å.

**Table 4:** Structure refinement

Resolution range (Å)	24.86–1.11
No. of reflections, working set	97727
No. of reflections, test set	4838
*R*_cryst_	0.155
*R*_free_	0.171
No. of non-H atoms	
Protein	2399
Ligands	36
Water	354
Total	2789
R.m.s.d., bond lengths (Å)	0.011
R.m.s.d., angles (°)	1.9
Average *B* factors (Å^2^)	
Protein	7.3
Ligands	11.4
Water	22.5
Ramachandran plot	
Most favoured (%)	98
Allowed (%)	2
Outliers (%)	0

## Data Availability

The structure of recombinant TaXyn10 has been deposited in the Protein Data Bank under accession code 31ct.
